# Levels and trends in mortality and causes of death among women of reproductive age in Bangladesh: Findings from three national surveys

**DOI:** 10.7189/jogh.13.07005

**Published:** 2023-08-25

**Authors:** Quamrun Nahar, Anadil Alam, Kaiser Mahmud, Shahnaj Sultana Sathi, Nitai Chakraborty, Abu Bakkar Siddique, Ahmed Ehsanur Rahman, Peter K Streatfield, Kanta Jamil, Shams El Arifeen

**Affiliations:** 1 International Centre for Diarrhoeal Disease Research, Dhaka, Bangladesh; 2 Independent Researcher, Dhaka, Bangladesh; 3 Data for Impact, University of North Carolina at Chapel Hill, Chapel Hill, USA; 4 Independent Consultant, Melbourne, Australia

## Abstract

**Background:**

Information on the mortality rate and proportional cause-specific mortality is essential for identifying diseases of public health importance, design programmes, and formulating policies, but such data on women of reproductive age in Bangladesh is limited.

**Methods:**

We analysed secondary data from the 2001, 2010, and 2016 rounds of the nationally representative Bangladesh Maternal Mortality and Health Care Survey (BMMS) to estimate mortality rates and causes of death among women aged 15-49 years. We collected information on causes of death three years prior to each survey using a country-adapted version of the World Health Organization (WHO) verbal autopsy (VA) questionnaire. Trained physicians independently reviewed the VA questionnaire and assigned a cause of death using the International Classification of Diseases (ICD) codes. The analysis included mortality rates and proportional mortality showing overall and age-specific causes of death.

**Results:**

The overall mortality rates for women aged 15-49 years decreased over time, from 190 per 100 000 years of observation in the 2001 BMMS, to 121 per 100 000 in the 2010 BMMS, to 116 per 100 000 in the 2016 BMMS. Age-specific mortality showed a similar downward pattern. The three diseases contributing the most to mortality were maternal causes (13-20%), circulatory system diseases (15-23%), and malignancy (14-24%). The relative position of these three diseases changed between the three surveys. From the 2001 BMMS to the 2010 BMMS and subsequently to the 2016 BMMS, the number of deaths from non-communicable diseases (e.g. cardiovascular diseases and malignancies) increased from 29% to 38% to 48%. Maternal causes led to the highest proportion of deaths among 20-34-year-olds in all three surveys (25-32%), while suicide was the number one cause of death for teenagers (19-22%). Circulatory system diseases and malignancy were the two leading causes of death for older women aged 35-49 years (40%-67%).

**Conclusions:**

There was a gradual shift in the causes of death from communicable to non-communicable diseases among women of reproductive age in Bangladesh. Suicide as the primary cause of death among teenage girls demands urgent attention for prevention.

Governments need timely and reliable statistics on mortality and causes of death to make effective public health decisions [1]. The most reliable source of mortality data are a fully functional civil registration and vital statistics (CRVS) system, which regularly captures births and deaths in a country [2]. However, only one-third of countries globally have CRVS systems able to regularly capture deaths and produce reliable cause of death statistics. In the remaining two-thirds of countries, deaths are not routinely registered nor medically certified, resulting in a lack of reliable information on underlying causes [2].

Bangladesh is a lower-middle-income country in South Asia with a population of 165 million [3], with an estimated three million births and 0.9 million deaths annually [4]. It is undergoing a rapid demographic and epidemiological transition, with an increase in the older population and a shifting disease burden from infectious communicable diseases to chronic non-communicable diseases (NCDs) [5]. Most deaths in the country are attributed to NCDs [5]. Analysis of data from Matlab – a rural area in Bangladesh with a demographic surveillance system that has collected information on the causes of death since 1966 – showed a substantial change in the mortality pattern from acute, infectious, and parasitic diseases to non-communicable, degenerative, and chronic disease over the past 20 years [6,7].

Bangladesh has no national system for registering deaths and determining their underlying causes [8], and its mortality estimates mainly come from censuses, sample surveys, and a sample registration system. The Bangladesh Bureau of Statistics (BBS) maintains a nationally representative Sample Vital Registration System (SVRS) that records causes of death based on information collected by a lay reporting system, but the accuracy of information collected through this system remains questionable [9].

One practical method for ascertaining cause of death is verbal autopsy (VA), a systematic approach to assess probable cause of death based on an interview with the next of kin or other caregivers who were present at the time of death or who are knowledgeable about the events leading up to it. VA is particularly useful in settings where deaths occur outside of hospitals, and can seemingly provide cause of death information that, at the population level, is similar to death certification in high-quality hospitals [10-12]. VA was previously used in Bangladesh to determine causes of death, including maternal deaths [13-15], childhood deaths [16-25], and adult female deaths [26]. Here we examined the trends in mortality rates and causes of death among women of reproductive age using data from three nationally representative surveys conducted in Bangladesh.

## METHODS

We performed a secondary analysis of data collected by the 2001, 2010, and 2016 rounds of the Bangladesh Maternal Mortality and Health Care Survey (BMMS), conducted by the National Institute of Population Research and Training (NIPORT) of the Government of Bangladesh with technical support from the International Centre for Diarrhoeal Disease Research, Bangladesh (icddr,b) and MEASURE Evaluation, University of North Carolina Chapel Hill, with the financial support from the United States Agency for International Development (USAID).

Each round of BMMS was conducted with a nationally representative sample with a three-stage cluster sampling to measure the maternal mortality ratio (MMR) with a three-year recall period [27-29]. The surveys covered 99 202 households in 2001, 168 629 in 2010, and 298 284 in 2016 [27-29] (**Table 1**).

**Table 1:** Survey time, number of interviews completed and number of women’s deaths captured in 2001, 2010, and 2016 BMMS

	2001 BMMS	2010 BMMS	2016 BMMS
**Data collection**	**January to June 2001**	**January to August 2010**	**August 2016 to February 2017**
Number of households interviewed	99 202	168 629	298 284
Number of ever-married women aged 13-49 years interviewed	103 796	175 621	321 214
Number of deaths captured among females aged 13-49 years	928	901	1524
Number of deaths captured among females aged 15-49 years	889	877	1478
Number of deaths captured among females aged 15-49 years in the three years preceding the survey (unweighted)	673	768	1254

In each survey, trained female interviewers (with at least a bachelor’s degree) visited the households and administered a structured questionnaire to collect information on household members and household deaths that happened in the three previous years, as well as information on the age, sex, and date of death of each deceased person. Deaths of women aged 13-49 were subsequently followed up by a separate group of trained female interviewers using the WHO-structured VA questionnaire adapted to the context of Bangladesh through expert consultations and translated into Bangla [30,31]. The VA respondent was the member of the household who knew the most about the deceased person and/or was present during the last illness and/or death.

Two physicians trained in reviewing VA forms and assigning a cause of death using the International Classification of Diseases, 10th Revision (ICD-10) classification [32] independently reviewed the VA forms and determined the cause of death. If disagreements occurred, the case was reviewed by a third physician, after which any two of three causes of death that matched from three physician reviews were considered as the final cause of death. In the case of disagreement, the cause of death was considered “undetermined”. Though VA was performed for all deaths reported among women aged 13-49 years at the time of death, we only present the underlying causes of death for women aged 15-49 years. Additional details on the methodology and assignment of causes of death are available in the BMMS reports [27-29].

### Analysis plan

We aimed to determine the mortality rates and proportional mortality showing overall and age-specific causes of death. We calculated the adult mortality rate for women of reproductive age (15-49 years) as the number of deaths in the three years preceding the survey to the person-years lived in the same period expressed per 100 000 women aged 15-49 years (person-years lived by the specific age groups for age-specific mortality). We calculated proportional mortality (%) by dividing the number of deaths attributed to a specific cause by the total number of deaths for which a VA was carried out. We grouped the ICD-10 codes into eight categories: maternal diseases, infectious diseases, malignancies, circulatory system diseases, suicide, other unnatural deaths, miscellaneous, and undetermined causes of death for which cause of death was not possible to assign or for which the reviewing physicians could not agree on a single cause of death.

We carried out a z test for proportions at a 95% confidence interval (CI) to assess the statistically significant difference (*P* < 0.05) between two proportions (between 2001 and 2010 or 2010 and 2016). The test statistic was:



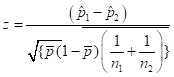


where *p̅* is the pooled mean. When comparing the significance of the difference between two mortality rates (2001 and 2010 or 2010 and 2016), we computed 95% CIs for both rates.

Since the data were from national household surveys which employed complex sample designs, including multistage sampling, stratification, and clustering, the sampling errors of the survey estimates cannot be computed using the formulae found in standard statistical method. The formula for calculating standard error and the corresponding 95% CI for each death is:



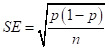


where *p* is the estimate of the sample (mortality rate) and *n* is the number of exposures.

We considered the design effect when calculating 95% confidence intervals to adjust the required sample size for survey data (2001 BMMS = 1.2, 2010 BMMS = 1.3, 2016 BMMS = 1.4).

The formula for 95% CI was *CI* = (*p* ± 1.96 × SE ± *deff*), where *p* is the mortality rate per 100 000 population. We used household weights to obtain unbiased estimates at the national level. We analysed the data using Stata version 15 (StataCorp LLC, Houston, TX, USA).

### Ethics approval

We used publicly available data sets from NIPORT and MEASURE Evaluation [33,34] and obtained the approval for secondary analysis from the MEASURE Evaluation.

## RESULTS

### Mortality rates

**Figure 1:**
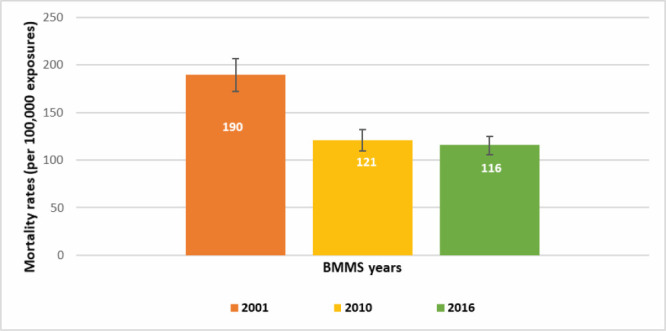
Mortality rates (per 100 000 years of observations) among women of reproductive age (15-49 years) in the 2001, 2010, and 2016 BMMS. y-axis shows mortality rates per 100 000 exposures and x-axis shows BMMS years.

The mortality rate of women (15-49 years) was 116 per 100 000 years of observations in the 2016 BMMS (95% CI = 106-125) compared with 121 per 100 000 years of observations in the 2010 BMMS (95% CI = 110-132) and 190 per 100 000 years of observations in the 2001 BMMS (95% CI = 172-207). We found a significant reduction in the mortality rate between the 2001 BMMS and the 2010 BMMS (**Figure 1**); however, the reduction between the 2010 BMMS and the 2016 BMMS was not statistically significant at a 95% confidence interval.

**Figure 2:**
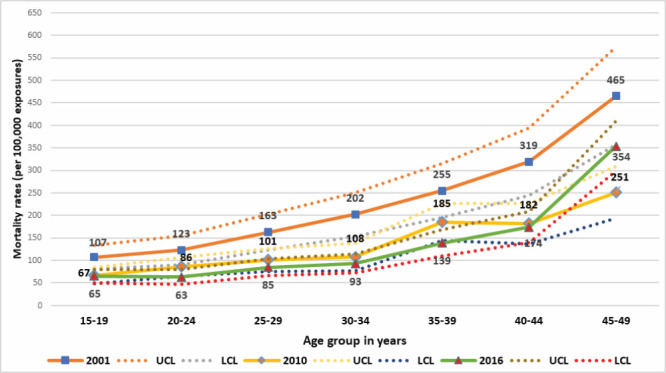
Age-specific mortality rates for women aged 15-49 years per 100 000 years of observations in the 2001, 2010, and 2016 BMMS. y-axis shows mortality rates per 100 000 exposures and x-axis shows age group in years.

The age-specific mortality rates showed similar patterns of decrease in all age groups in the three surveys, except for the oldest age group (45-49 years) (**Figure 2**), for which the mortality rate increased in 2016 (354 per 100 000) compared to 2010 (251 per 100 000).

**Figure 3:**
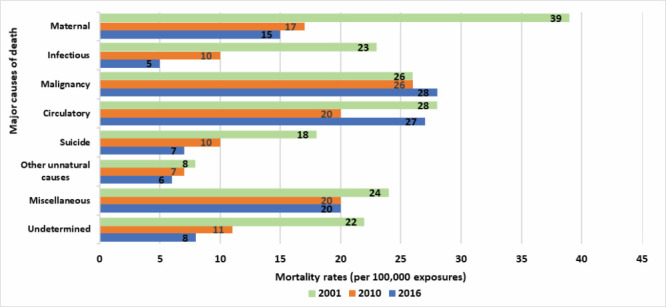
Mortality rates per 100 000 years of observations by major causes of death in the 2001, 2010, and 2016 BMMS. y-axis shows major causes of death and x-axis shows mortality rates per 100 000 exposures.

Among women aged 15-49 years in the three surveys, cause-specific mortality rates were the highest for maternal causes, circulatory system diseases, and malignancies (**Figure 3**). In the 2016 BMMS, malignancies had the highest mortality rates (28 per 100 000), followed by circulatory system diseases (27 per 100 000) and maternal causes (15 per 100 000). The pattern was similar in the 2010 BMMS (malignancies: 26 per 100 000, circulatory diseases: 20 per 100 000, maternal causes: 17 per 100 000). However, in the 2001 BMMS, maternal causes had the highest mortality rates (39 per 100 000), followed by circulatory diseases (28 per 100 000) and malignancies (26 per 100 000).

When comparing the three surveys, mortality rates from most of the causes decreased over time, except for deaths due to malignancies and circulatory system diseases. We observed the highest, statistically significant decrease in death rates due to infectious diseases (57%) and maternal causes (56%) between the 2001 BMMS and the 2010 BMMS.

The mortality rate due to infectious diseases continued to decrease significantly at the same pace between the 2010 BMMS and the 2016 BMMS (54%), while the decline for maternal causes was slow (12%) during the same period. The mortality rate due to suicide declined first between the 2001 BMMS and the 2010 BMMS (45%) and further in the 2016 BMMS (33% decline compared to the 2010 BMMS). The primary means of suicide was hanging, followed by ingestion of chemical poisons and pesticides.

However, deaths due to circulatory diseases decreased between the 2001 BMMS and the 2010 BMMS (31%), but increased in the 2016 BMMS, returning to almost the same level as the 2001 BMMS rate (36% increase between the 2010 BMMS and the 2016 BMMS). The mortality rate from different malignant causes remained static (26-28 per 100 000) during the 15 years between the three surveys.

Under the miscellaneous category, diabetes, hepatitis, and other liver diseases, renal failure, and chronic respiratory diseases were the main causes of death in the three surveys. The death rate due to miscellaneous causes decreased between the 2001 BMMS and the 2010 BMMS (17%) and remained static afterwards. The deaths categorised as “undetermined” decreased from the 2001 BMMS to the 2010 BMMS (51%) and further in the 2016 BMMS (29%).

Mortality rates due to different causes varied by age groups (Table S1 in the **Online Supplementary Document**). Although the mortality rate due to suicide was the highest among adolescents and young women aged 15-29 years, the death rate due to maternal causes increased beginning at age 20 and decreased after age 29 in 2001 and 2010 surveys (with an increase at 40-44 years), and after age 39 in the 2016 survey. Death rates from circulatory diseases and malignancies increased sharply with age, especially after 34 years of age. Death rates due to other unnatural causes, such as injuries, drowning, snakebites, and other causes showed no clear age pattern. Death rates due to miscellaneous and undetermined causes for which a specific cause could not be determined increased moderately with age. These patterns did not change among the three surveys.

### Proportional mortality by cause of death

In the 2016 BMMS, malignancies caused the most deaths (24%), followed by circulatory system diseases (23%) and maternal causes (13%). We found a similar pattern in the 2010 BMMS (malignancy: 22%, circulatory system diseases: 16%, maternal causes: 14%). By contrast, in the 2001 BMMS, maternal causes led to the highest number of deaths (20%), followed by circulatory system diseases (15%), and malignancies (14%). The proportion of deaths due to suicide changed significantly in 2016 compared to the 2010 BMMS (*P* = 0.03). Other unnatural causes did not change during the 15 years between the three surveys and accounted for 4% to 6% of total deaths, respectively (**Table 2**).

**Table 2:** Proportional mortality n (%) among women aged 15-49 years in the three years preceding the 2001, 2010, and 2016 BMMS surveys, stratified by cause of death

Causes of death	2001 BMMS	2010 BMMS	2016 BMMS	*P*-value (between 2001 and 2010)	*P*-value (between 2010 and 2016)
Maternal	139 (20.4)	104 (14.2)	160 (13.1)	0.00*	0.49
Infectious	84 (12.4)	61 (8.3)	49 (4.0)	0.01*	0.00*
Malignancy	94 (13.9)	159 (21.6)	299 (24.4)	0.00*	0.16
Circulatory disease	101 (14.9)	119 (16.2)	282 (23.0)	0.50	0.00*
Suicide	65 (9.6)	62 (8.4)	72 (5.9)	0.43	0.03*
Other unnatural causes	30 (4.4)	43 (5.8)	67 (5.5)	0.23	0.78
Miscellaneous	87 (12.9)	123 (16.7)	213 (17.4)	0.04*	0.69
Undetermined	79 (11.6)	66 (9.0)	81 (6.7)	0.00*	0.00*
N	679 (100)	737 (100)	1223 (100)		

*Proportional mortality (%) by cause of death was compared between 2001 and 2010 BMMS and between 2010 and 2016 BMMS. *P* < 0.05 denotes statistical significance.

**Figure 4:**
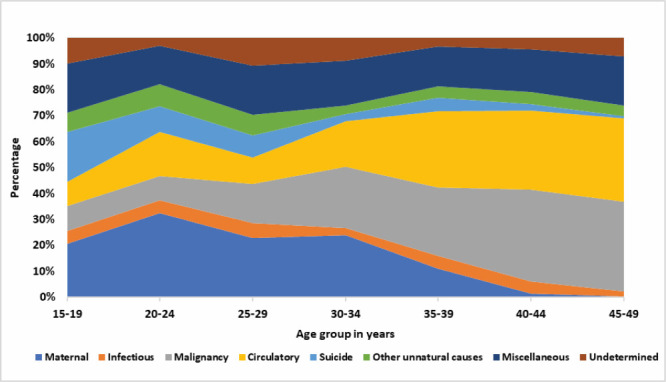
Proportional distribution of different causes of death by different age groups, 2016 BMMS. y-axis shows percentage (Proportional distribution of different causes of death**)** and x-axis shows age group in years.

The proportional distribution of the different causes varied greatly by age groups (**Figure 4**). Maternal deaths contributed to most deaths for age groups up to age 34 (21-33%), while circulatory system diseases (29-32%) and malignancies (26-35%) caused the most deaths among women over 35 years. Suicide was the second most common cause of death among teenagers (19%) after maternal causes (20%).

Although maternal deaths contributed the most to deaths among 20-34-year-olds in the three surveys, unnatural deaths (suicide and other unnatural deaths) were the topmost killer for teenagers (Table S2 in the **Online Supplementary Document**). In 2001, 28% of teenagers had unnatural deaths, increasing to 36% in the 2010 BMMS and then decreasing to 27% in the 2016 BMMS.

**Figure 5:**
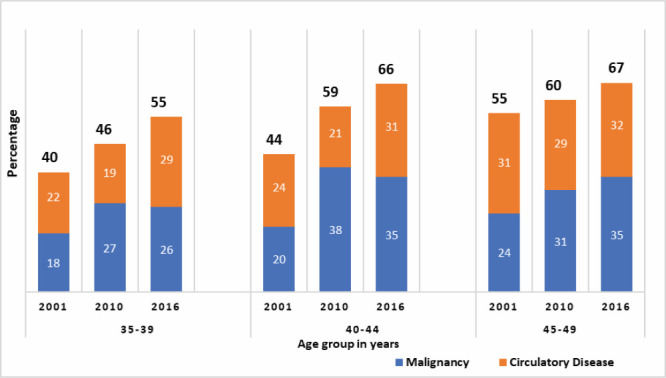
Proportion of deaths due to malignancy and circulatory system diseases for women aged 35-49 years in the 2001, 2010, and 2016 BMMS. y-axis shows the percentage (proportion of deaths due to malignancy and diseases of the circulatory system) and x-axis shows age group in years.

Circulatory system diseases and malignancies were the two leading causes of death for older women aged 35-49 years; this proportion increased with age and over time (**Figure 5**). These two diseases together accounted for 40-55% of the total deaths for 35-49-year-old women in the 2001 BMMS, 46-60% in the 2010 BMMS, and 55-67% in the 2016 BMMS. These increases were consistent for each of the two diseases.

We found little variation in the type of malignancy (by location) for women who died from malignancies from between the three surveys. Cancer of the gastrointestinal tract, including the liver, contributed the most to the number of deaths (30-37%), followed by cancers of the reproductive organs, including breast and cervical cancer (27-29%), blood and lymphatic system cancers (10-28%), and other cancers (13-26%) (data not shown).

## DISCUSSION

Using data from three nationally representative surveys, we examined mortality rates and causes of death among women of reproductive age in Bangladesh over a 15-year period. We observed a major downward shift in the overall and age-specific mortality for all age groups during the reporting period covered by the three surveys. This is consistent with previous studies that showed a consistent decline in mortality for both men and women in all age groups nationally [35,36] and in a rural health and demographic surveillance site in Bangladesh [37].

Mortality for both sexes started to decline in the country in the mid-20th century, intensifying in recent decades. This may be due to improvements in the overall socioeconomic status of the general population, along with improvements in the use of health services in both the public and private sectors. Life expectancies have also increased for both women and men during the same period. From 2001 to 2021, the average life expectancy increased from 67 to 74 years for women and from 66 to 71 years for men [38].

Maternal causes (13%-20%), circulatory system diseases (15%-23%), and malignancy (14%-24%) contributed the most to mortality in the three surveys, but their relative position changed over time. There was a major shift in the causes of women’s deaths between the 2001 and 2010 surveys; deaths due to maternal causes decreased and deaths due to NCDs (like circulatory system diseases, malignancies, and other chronic conditions) increased. The rise of deaths due to NCDs continued in the 2016 survey, and NCDs became a major cause of deaths. Circulatory system diseases and malignancies were the two leading causes of death for older women aged 35-49 years.

These findings are consistent with other studies documenting NCDs as the major cause of adult deaths, including women’s deaths in Bangladesh [5,13,35,39,40]. In a cohort of rural Bangladeshi women, Labrique et al. [39] recorded deaths that occurred between 2001-2007 and found that pregnancy-related (22%) contributed a major number of deaths, followed by deaths due to circulatory system diseases (19%) and malignancy (15%).

Like many other developing countries, Bangladesh is undergoing an epidemiological transition from communicable diseases to NCDs, which contribute more than two-thirds of annual deaths in Bangladesh (i.e. over half a million per year), according to the WHO [5]. The most common NCDs are cardiovascular diseases, diabetes mellitus, malignancy, and chronic respiratory diseases [41].

Considering the huge burden of NCDs, the Government of Bangladesh has undertaken several initiatives based on the WHO’s Global Action Plan for the Prevention and Control of NCDs 2013-2020 [42], with a number of national policies and strategies targeting specific NCDs. However, several gaps exist in the proper planning, implementation, and monitoring of those initiatives [43]. The country still lacks a comprehensive and integrated approach for NCD prevention and control, as well as coordination among the public, nongovernmental, and private sectors in urban and rural areas.

This lack of coordination is reflected in the high concentration of specialised services in urban areas, particularly at tertiary facilities. Although private providers play an important role in NCD-related service delivery, they are not systematically integrated within a comprehensive framework of programmes. Existing health services at the primary health care level are deficient, lacking an appropriately trained health workforce, necessary instruments for screening of NCDs and supply of essential NCD medicines, and tend to emphasise curative management rather than prevention [44].

Despite a significant decline in maternal mortality among all age groups between the 2001 and 2010 BMMS, maternal deaths stalled after 2010, as shown in the 2016 survey. The average annual rate of reduction of the MMR over the 15 years between 2001 and 2016 was 3.3%. However, to achieve the Sustainable Development Goal target of reducing the MMR from 170 to 105 per 100 000 live births by 2030, a yearly relative reduction rate of 7.4% for the MMR is required in Bangladesh. According to the 2016 BMMS, more than half of maternal deaths were caused by haemorrhage and eclampsia, most of which could have been prevented through simple and inexpensive measures.

A substantial proportion of women died due to unnatural causes including suicide in all three surveys. Although suicide deaths decreased significantly in the last survey compared with the previous two, deaths due to other unnatural causes increased substantially during the same period, one of which was road traffic accidents. Unnatural deaths, including suicide, continued to be the number one cause of death among teenage girls – one in every three teenage girls died untimely due to causes that were preventable.

Suicide is a complex, multi-faceted preventable public health problem that is under-researched in Bangladesh. A recent systematic review of 35 articles concluded that the actual rate of suicide in Bangladesh was unknown and the quality of existing data was a challenge [45]. Literature on suicide is dominated by newspaper reporting, with only a handful of studies conducted to characterise suicide in Bangladesh. One study from 1996-1997 found that suicide was an important cause of death among women aged 10-50 years [46]. Another study conducted in 2003 found that suicide was the fourth highest injury-related cause of death after drowning, road traffic accident and fall, and the leading cause of injury-related deaths among adolescents aged 10-19 years, with slightly higher risk of suicide among women compared to men [47]. The overall suicide rate among adult women was found to be 8.2 per 100 000 in this study compared to 6.5 per 100 000 among men. Another study conducted in Matlab, a rural area of Bangladesh, found an average annual death rate due to suicide among women 15-44 years old of 13 per 100 000 during 1982-1998 [13].

Suicide is a criminal offence in the legal system in Bangladesh and all suicide cases are mandated to be reported to the police. Consequently, there is a strong likelihood of underreporting of suicidal deaths in any suicide-related study. In this study, however, information collected on the causes of death via VA was later reviewed by expert physicians and the likelihood of misdiagnosis of suicidal deaths was likely low. We found that the suicide rate among women was 7-18 per 100 000 years of observation, which is comparable to previous studies and therefore confirms that suicide is a major public health concern that cannot be ignored when designing health programmes and formulating policies.

### Strengths and challenges

We based our study on data from three nationally representative surveys, and the sample size for each was large enough to draw conclusions at the national level. The three surveys were comparable in design, providing a unique opportunity to not only report mortality rates and causes of death within the years in which the surveys took place, but also to highlight changes in overall and age-specific mortality and causes of death over time.

The VA questionnaires used were based on the standard WHO tool and adapted by a group of experts, many of whom participated in conducting all three surveys, making the results across the surveys comparable. Highly trained interviewers were assigned to conduct the VA interviews, and the review physicians had previous experience in reviewing VA forms. These factors contributed to ensuring that the resulting cause of death data was of high quality.

Notably, 7%-12% of the deaths reported in this study could not be assigned a cause of death, leaving the causes “undetermined”. Although the VA interview involved collecting information on the signs and symptoms leading up to death, the respondents may have gaps in their knowledge or be affected by recall bias. As highlighted in studies such as the one by Mwanyangala et al. [48], the age at death, respondents’ level of education, place of death, and the respondents’ relationship with the decedents can all be associated with undetermined causes of death.

## CONCLUSIONS

Bangladesh has achieved commendable success in the health and development sectors over the past four decades, seeing decreasing mortality rates in all age groups and increased longevity of both men and women. However, the country is approaching the advanced stages of its epidemiological transition, creating several challenges for the health sector. The elderly population has grown and the burden of NCDs has increased, as has the proportion of deaths due to unnatural causes. Of these deaths due to unnatural causes, suicide – especially among young women – is particularly challenging. Moreover, maternal mortality rates initially improved and then stalled after 2010.

The government’s strategies for addressing these challenges should build on its past successes. Future health sector programs should aim to confront rising morbidity and mortality due to NCDs and unnatural deaths. In particular, national policies to target the rise of NCDs should be underpinned by an integrated framework for NCD prevention and control, including coordination between the public, nongovernmental, and private sectors in both urban and rural areas. By ensuring that the health workforce is appropriately trained and equipped, Bangladesh can continue on its trajectory towards reducing its mortality rates.

## Additional material


Online Supplementary Document
